# Protein Phosphatase 2A as a Therapeutic Target in Acute Myeloid Leukemia

**DOI:** 10.3389/fonc.2016.00078

**Published:** 2016-04-06

**Authors:** Elena Arriazu, Raffaella Pippa, María D. Odero

**Affiliations:** ^1^Hematology/Oncology Program, Center for Applied Medical Research (CIMA), University of Navarra, Pamplona, Spain; ^2^Centre for Gene Regulation and Expression, University of Dundee, Dundee, UK; ^3^Department of Biochemistry and Genetics, University of Navarra, Pamplona, Spain; ^4^Instituto de Investigación Sanitaria de Navarra (IdiSNA), Pamplona, Spain

**Keywords:** PP2A, SET, AML, FTY720, OP449

## Abstract

Acute myeloid leukemia (AML) is a heterogeneous malignant disorder of hematopoietic progenitor cells in which several genetic and epigenetic aberrations have been described. Despite progressive advances in our understanding of the molecular biology of this disease, the outcome for most patients is poor. It is, therefore, necessary to develop more effective treatment strategies. Genetic aberrations affecting kinases have been widely studied in AML; however, the role of phosphatases remains underexplored. Inactivation of the tumor-suppressor protein phosphatase 2A (PP2A) is frequent in AML patients, making it a promising target for therapy. There are several PP2A inactivating mechanisms reported in this disease. Deregulation or specific post-translational modifications of PP2A subunits have been identified as a cause of PP2A malfunction, which lead to deregulation of proliferation or apoptosis pathways, depending on the subunit affected. Likewise, overexpression of either SET or cancerous inhibitor of protein phosphatase 2A, endogenous inhibitors of PP2A, is a recurrent event in AML that impairs PP2A activity, contributing to leukemogenesis progression. Interestingly, the anticancer activity of several PP2A-activating drugs (PADs) depends on interaction/sequestration of SET. Preclinical studies show that pharmacological restoration of PP2A activity by PADs effectively antagonizes leukemogenesis, and that these drugs have synergistic cytotoxic effects with conventional chemotherapy and kinase inhibitors, opening new possibilities for personalized treatment in AML patients, especially in cases with SET-dependent inactivation of PP2A. Here, we review the role of PP2A as a druggable tumor suppressor in AML.

## Introduction

Acute myeloid leukemia (AML) is a heterogeneous clonal disorder of hematopoietic progenitor cells, which predominantly affects elderly adults. It is characterized by a differentiation blockade of the myeloid hematopoietic progenitor cells accompanied by uncontrolled proliferation. As a consequence, immature cells accumulate in bone marrow and peripheral blood. With the exception of acute promyelocytic leukemia, therapy for AML is not targeted, and the intensity of therapy is driven by the prognostic subgroup. Cytogenetic and molecular genetic aberrations have been postulated as the most powerful markers for survival and therapy response in AML, with patients classified into favorable, intermediate or poor prognosis (1, 2). Although major improvements have been achieved in the overall survival of adult cases ≤60 years, most of the patients are older than 60 years, and in this group only 5–15% are cured (3, 4). Furthermore, the outcome in older patients, who are unable to receive intensive chemotherapy without unacceptable side effects, remains dismal, with a median survival of only 5–10 months (5–7). Therefore, it is necessary to develop more effective treatment strategies for this disease.

The uncontrolled growth of transformed cells is caused by the deregulation of multiple cellular pathways that are involved in normal growth control (8). Reversible phosphorylation is one of the mechanisms that cells use to maintain normal homeostasis, and is involved in several processes, such as proliferation, apoptosis, and differentiation; hence, kinases and phosphatases act as important checkpoint regulators (9). Numerous studies have focused on studying the aberrant kinase behavior in AML; however, although phosphatases are also essential to maintain the correct homeostasis, their role in AML has not been fully considered. In this review, we will focus on the role of protein phosphatase 2A (PP2A), inactivation of which is a recurrent event in AML, as a druggable tumor suppressor.

## Protein Phosphatase 2A

**Figure 1:**
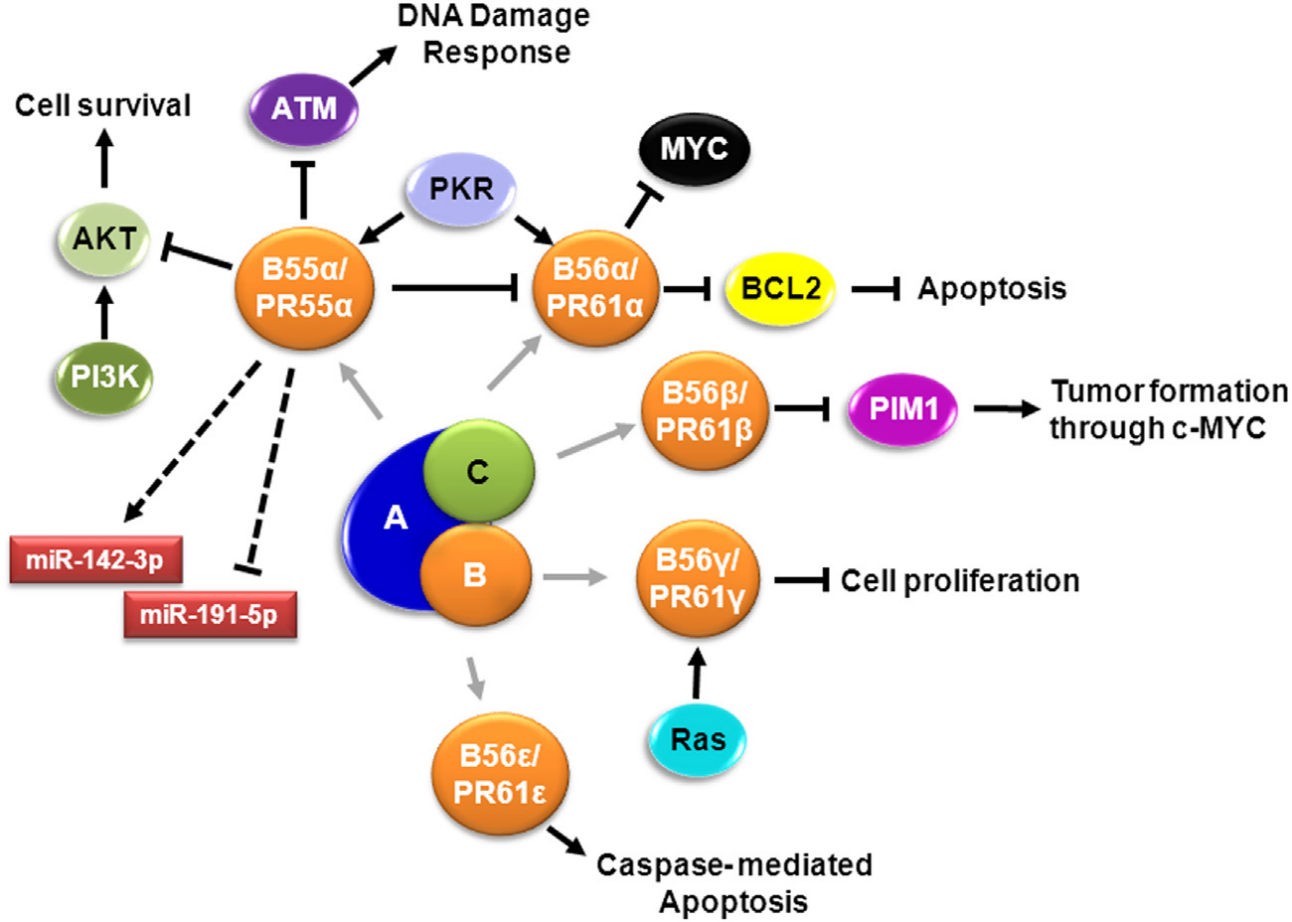
**Signaling pathways involving PP2A in AML**. Schematic representation of known PP2A complexes involving different B regulatory subunits in AML cells. Cell survival is regulated by PR55α/B55α – mediated dephosphorylation of AKT (15, 16). PR55α/B55α also supports expression of miR-142-3p and suppresses expression of miR-191-5p, relevant miRNAs in AML (17). DNA damage response is impaired by dephosphorylation of ATM by PP2A-PR55α/B55α, which translocate to the nucleus by PKR (18). Extracellular survival signals activate SRC that suppresses the B subunit; when SRC is suppressed, PR55α/B55α is expressed, resulting in dephosphorylation of PKCα and suppression of PR61α/B56α protein expression, with concomitant induction of MYC (19). Apoptosis is regulated by activation of PR61α/B56α by PKR leading to dephosphorylation of BCL2 (20). PR61β/B56β regulates PIM1 contributing to tumor formation (21). PP2A-B56γ function in G2 is crucial to sustain normal G0/G1 control and this G2 PP2A function involves modulation of endogenous RAS signaling (22). PR61ϵ/B56ϵ, which is downregulated in AML, controls caspase-mediated apoptosis (23).

Protein phosphatase 2A, one of the main serine/threonine phosphatases in mammalian cells, is a tumor suppressor that regulates several essential functions and counteracts most of the kinase-driven intracellular signaling pathways (8, 10, 11) (Figure 1). PP2A inactivation occurs in several solid and hematological tumors, leading to sustained activation of survival pathways or inhibition of apoptotic pathways (12, 13). Studies with the potent tumor promoter okadaic acid (OA), which inhibits the enzymatic activity of PP2A, have contributed to our understanding of phosphatase functions (14).

Protein phosphatase 2A exists in two different forms: as a dimer and in a trimeric form (24). The dimeric form is known as the core enzyme and consists of a structural A subunit and a catalytic C subunit. These subunits are coded by two different genes, which may further generate two distinct isoforms: PP2A-A (*PPP2R1A*/Aα and *PPP2R1B*/Aβ) and PP2A-C (*PPP2CA*/Cα and *PPP2CB*/Cβ) (Table 1). The trimeric form is an active holoenzyme complex that consists of three subunits: the above-mentioned scaffold (PP2A-A) and catalytic (PP2A-C) subunits, and a regulatory B subunit (PP2A-B) (Figure 1; Table 1). In its heterotrimeric form, the structural subunit mediates the interaction between the catalytic subunit with a variety of regulatory PP2A-B subunits; whereas in the dimeric form, PP2A-A acts as a regulator by changing the catalytic specificity (25). Four unrelated families of regulatory PP2A-B subunits (B/PR55/B55, B′/PR61/B56, B′′/PR72, and B′′′/the striatins, STRN) have been identified, including at least 23 different alternative transcripts and spliced forms, which determine the substrate specificity and the intracellular location of the PP2A complex (Figure 1; Table 1) (12, 26, 27). The actual challenge is not only to explore the potential therapeutic value of PP2A activators (28–30), but also to identify the particular PP2A complexes affected in each disease, in order to develop more efficient therapeutic strategies (31).

**Table 1:** **PP2A subunits and reported alterations in AML**.

Family	Gene	Locus	Protein	Alterations reported in AML
A	PPP2R1A	19q13.41	PR65α/Aα	Downregulation (32)
				Oncogenic c-KIT mutations decrease protein levels (29)
	PPP2R1B	11q23.1	PR65β/Aβ	Downregulation. No good correlation between mRNA and protein (30)
				Downregulation (33). Data collected as part of the Cancer Genome Atlas (TCGA)
C	PPP2CA	5q31.1	PP2Acα/Cα	Downregulation in TP53 mutant AML cases (33). Data collected as part of the Cancer Genome Atlas (TCGA)
	PPP2CB	8p12	PP2Acβ/Cβ	
B	PPP2R2A	8p21.2	PR55α/B55α	Oncogenic c-KIT mutations decrease protein levels (29)
				Downregulation at protein level (16, 19)
				Downregulation (32)
	PPP2R2B	5q32	PR55β/B55β	High expression (33). Data collected as part of the Cancer Genome Atlas (TCGA)
				Somatic mutation (one AML case) [Data collected as part of the Cancer Genome Atlas (TCGA)]
	PPP2R2C	4p16.1	PR55γ/B55γ	Downregulation (33). Data collected as part of the Cancer Genome Atlas (TCGA)
	PPP2R2D	10q26.3	PR55δ/B55δ	
B′	PPP2R5A	1q32.3	PR61α/B56α	Oncogenic c-KIT mutations decrease protein levels (29)
	PPP2R5B	11q13.1	PR61β/B56β	Downregulation. Good correlation between mRNA and protein (30)
				High expression (33). Data collected as part of the Cancer Genome Atlas (TCGA)
	PPP2R5C	14q32.31	PR61γ/B56γ	Downregulation (30)
				Oncogenic c-KIT mutations decrease protein levels (29)
	PPP2R5D	6p21.1	PR61δ/B56δ	Oncogenic c-KIT mutations decrease protein levels (29)
	PPP2R5E	14q32.2	PR61ϵ/B56ϵ	Downregulation. Good correlation between mRNA and protein (23)
B′′	PPP2R3A	3q22.2	PR72/PR130	
	PPP2R3B	Yp11.32; Xp22.33	PR70/PR48	Downregulation (33). Data collected as part of the Cancer Genome Atlas (TCGA)
	PPP2R3C	14q13.2	G5PR	
B′′′	STRN	2p22.2	Striatin	
	STRN3	14q13-q12	Striatin3	
	STRN4	19q13.2	Striatin4	

The precise mechanism of assembly of active PP2A holoenzyme is still incompletely understood [reviewed in Ref. (34)]. The activity of PP2A can be regulated by post-translational modifications. Methylation and phosphorylation of residues from PP2A-C subunits modulate the formation of the complex. For instance, methylation of leucine 309 (L309) in the catalytic PP2A-C subunit, by the leucine carboxyl methyltransferase 1 (LCMT1), is indispensable for binding the PR55/B55 subunit, although it is not an essential requisite for other B families (34–36). Phosphorylation of tyrosine 307 (Y307) dramatically impairs PP2A phosphatase activity by inhibiting the interaction of PP2A-C with the PR55/B55 and PR61/B56αβγϵ subunits; whereas threonine 304 (T304) phosphorylation prevents the assembly of PR55/B55 to the core enzyme (34). Interestingly, increased phosphorylation of Y307 of PP2A-C is a common event in both cell lines and AML patient samples (30). Additionally, post-translational modification of B subunits can affect the sub-cellular localization of PP2A, influencing which proteins are targeted (9).

We and others have reported that PP2A inhibition is a recurrent event in AML, and that restoration of PP2A phosphatase activity by treatment with PP2A-activating drugs (PADs) has antileukemic effects in both c-KIT wild-type (c-KIT^−^) and c-KIT mutated (c-KIT^+^) AML cells, inducing cell growth arrest and caspase-dependent apoptosis (12, 13, 29, 30, 33, 37). Furthermore, we have shown that PADs can be used alone or in association with either kinase inhibitors or traditional chemotherapy in AML, suggesting that PP2A rescue could represent an innovative therapeutic target in this disease (29, 30, 37–41).

## Mechanisms of PP2A Inactivation in AML

Transformed cells display a wide variety of mechanisms to inactivate PP2A. Several somatic mutations in PP2A subunits have been described in different types of tumors, such as melanoma, lung, colon, and breast cancers (31, 42–47). Mutations of structural PP2A-Aα and/or PP2A-Aβ subunits cause a defective binding of B and C subunits, inhibiting PP2A activity and contributing to cell transformation (44, 45). However, this seems to be an uncommon mechanism in AML. Our analysis of data from the Cancer Genome Atlas Research Network (https://tcga-data.nci.nih.gov/tcga), which analyzed the genomes of 200 patients with AML (50 with the use of whole-genome sequencing and 150 with the use of whole-exome sequencing), show that only one case has somatic mutations in PPP2R2B, the gene encoding the PP2A subunit PR55β (48).

We and other groups have shown that deregulation of some PP2A subunits, deregulated expression of the endogenous PP2A inhibitors SET or cancerous inhibitor of protein phosphatase 2A (CIP2A), or overexpression of SETBP1, contribute to PP2A inhibition in AML (28, 30, 40, 49, 50).

### Deregulation of PP2A Subunits

Alterations of the PP2A subunits have been found in AML with different genetic backgrounds, contributing to the malignant process (Table 1). Downregulation of the Aβ subunit is a common event in AML (30). Most cellular PP2A holoenzymes contain the Aα isoform of the scaffold subunit, but a small fraction (10%) contain a second isoform termed Aβ. As indicated above, mutations that disrupt the ability of Aβ to form holoenzymes *in vitro* were identified in several types of cancer, but Sablina et al. provide the first hard evidence that loss of functional Aβ due to these cancer-associated mutations contributes to transformation (8, 51). Suppression of PP2A Aβ permits immortalized human cells to achieve a tumorigenic state through the deregulation of RaIA GTPase activity. Cancer-associated Aβ mutants fail to reverse this tumorigenic phenotype, indicating that these mutants function as null alleles (51). In addition, both Aα mutants and Aα downregulation lead to a functional haploinsufficiency that seems to induce human cell transformation by activating the AKT/PI3K signaling pathway (51, 52). However, it is likely that different sets of genetic aberrations during tumor formation require the loss of different PP2A holoenzyme complexes for the tumor progression, and this would involve the regulatory subunits, which are playing a key role directing PP2A to dephosphorylate and regulate key tumor suppressors or oncogenes (26).

Altered expression of the scaffold as well as the regulatory subunits has been reported in AML patients with c-KIT mutations, which is associated with poor outcome in AML (Table 1) (32, 53). c-KIT is a type 3 receptor tyrosine kinase, the activation of which induces proliferation, differentiation, and survival. Oncogenic c-KIT mutations reduce PP2A activity by decreasing protein levels of PR65α, PR55α, PR61α, PR61δ, and PR61γ (Table 1) (29). c-KIT-mediated growth and survival may be prevented by overexpressing PP2A-Aα in myeloid c-KIT^+^ cells, suggesting that restoration of PP2A activity in c-KIT^+^ AML patients may represent a good therapeutic strategy to overcome drug resistance (29).

The PP2A-PR55/B55 family consists of four different isoforms (α, β, γ, and δ) associated with several core-signaling pathways, including ARF/MDM2/p53, PI3K/AKT, Raf/MEK/ERK, TGFBR1/TGF-β, and Wnt/β-catenin (54), and the regulation of the cell cycle and mitosis (55, 56). In AML cells, PP2A-PR55α dephosphorylates AKT on threonine 308 (T308) (15, 16). Interestingly, increased phosphorylation of AKT correlates with poor outcome in AML (57). Ruvolo et al. quantified the expression of several transcripts in 30 newly diagnosed patients with AML, and found that the expression of *PPP2CA*, *PPP2CB* (catalytic C subunits), and *PPP2R2A* (regulatory PR55α subunit) was elevated in blast cells. However, when they looked at protein expression, the levels of PR55α were low in the blast cells from the AML patients, suggesting different rates of translation, degradation, cleavage, or post-translational inactivation (Table 1) (16). As expected, they saw a link between expression of PR55α and AKT dephosphorylation. This suggests that strategies to promote PR55α inactivation of AKT may be useful for the therapy of AML (16). Apart from its function regulating cell survival, PR55α has been discovered as a modulator of the expression of microRNA relevant for AML, such as miR-142-3p and miR-142-5p. These miRs are found to be mutated in 2% of AML patients, although the mechanisms and the implications are still unknown (17, 19). In addition, a recent study demonstrated that PR55α is also implicated in DNA damage response in AML. Cheng et al. found that the double-stranded RNA-activated protein kinase PKR activates PP2A by promoting the nuclear localization of PR55α/B55α. Activated PP2A in turn antagonizes autophosphorylation and activation of ATM and its association with downstream targets, preventing DNA damage response and contributing to transformed phenotype (18). Significantly, PKR is involved in AML progression (58, 59), and high PKR expression is associated with poor overall survival and shortened remission duration for AML patients (18).

Several members of the PR61/B56 family of regulatory PP2A subunits appear to have a main role in directing PP2A potential tumor-suppressive activity (21, 60–63). The PP2A-PR61/B56 regulatory family has five different isoforms (α, β, γ, δ, and ϵ) (49), which can bind directly to the core enzyme and be regulated by phosphorylation from kinases (20, 64). In acute lymphoblastic leukemia cells, it has been described that PKR promotes the mitochondrial localization of PP2A–PR61α, leading to BCL2 dephosphorylation and inactivation and, therefore, contributing to apoptosis (20). Downregulation of PP2A–PR61β and PP2A–PR61γ seems to be a common event in AML cases, leading to the inactivation of PP2A, and consequently contributing to malignant cell proliferation (Table 1) (30). PP2A–PR61β has been reported as a tumor suppressor that negatively regulates PIM1 protein kinase, enhancing the ability of c-MYC to induce lymphomas (21). PP2A–PR61γ plays a crucial role in cell proliferation (65), in part due to dephosphorylation of p53 (66). In accordance with this, it has been described that suppression of PR61γ expression contributes to the experimental transformation of human cells (31, 60). The function of PP2A–PR61γ in G2 is crucial to sustain normal G0/G1 control, and this G2 PP2A function involves modulation of endogenous RAS signaling (22). Therefore, loss of PR61β/B56β and PR61γ/B56γ could be playing a role in AML development, contributing to deregulate the correct PP2A function.

PR61ϵ/B56ϵ is recurrently downregulated at mRNA and protein level in AML patients (Table 1), contributing to cell proliferation (23). This regulatory subunit is involved in multiple signaling pathways and plays critical roles during early development (67–69). Moreover, PR61ϵ is an essential regulator of apoptosis (70), and acts as a negative regulator of MAP4K3, mediating its ability to signal to mTORC1 (71). In AML cells, PR61ϵ impairs cell proliferation, induces caspase-dependent apoptosis, affects the activation status of AKT, and reduces the colony-forming ability of the leukemic cells. Moreover, there is a good correlation between PR61ϵ downregulation and p53 levels, suggesting that the molecular effects of this B subunit in AML could occur, at least in part, via p53 (23). These results indicate that PR61ϵ downregulation has relevance in AML, and could allow distinguishing a subgroup of patients who could benefit from receiving future treatments with PP2A activators.

Aberrations of the other two families of regulatory PP2A-B subunits have been involved in several solid tumors (31, 72–75) but not in AML. Therefore, further studies are necessary to clarify the importance of these PP2A subunits in hematological malignancies.

### SET/I2PP2A

The SET protein, also named I2PP2A (Inhibitor 2 of PP2A), TAF-1β or PHAP1, is a potent endogenous inhibitor of PP2A with an important role in myeloid leukemias (76). SET was first identified as an oncogene fused with the nucleoporin NUP214 (CAN) in acute undifferentiated leukemia (77), and soon after, it was described as a PP2A inhibitor (78). This protein is located mostly in the nucleus, and it is implicated in many cell processes, such as DNA replication, chromatin remodeling, gene transcription (79, 80), DNA repair (81), differentiation (82), migration (83), and cell-cycle regulation (84).

**Figure 2:**
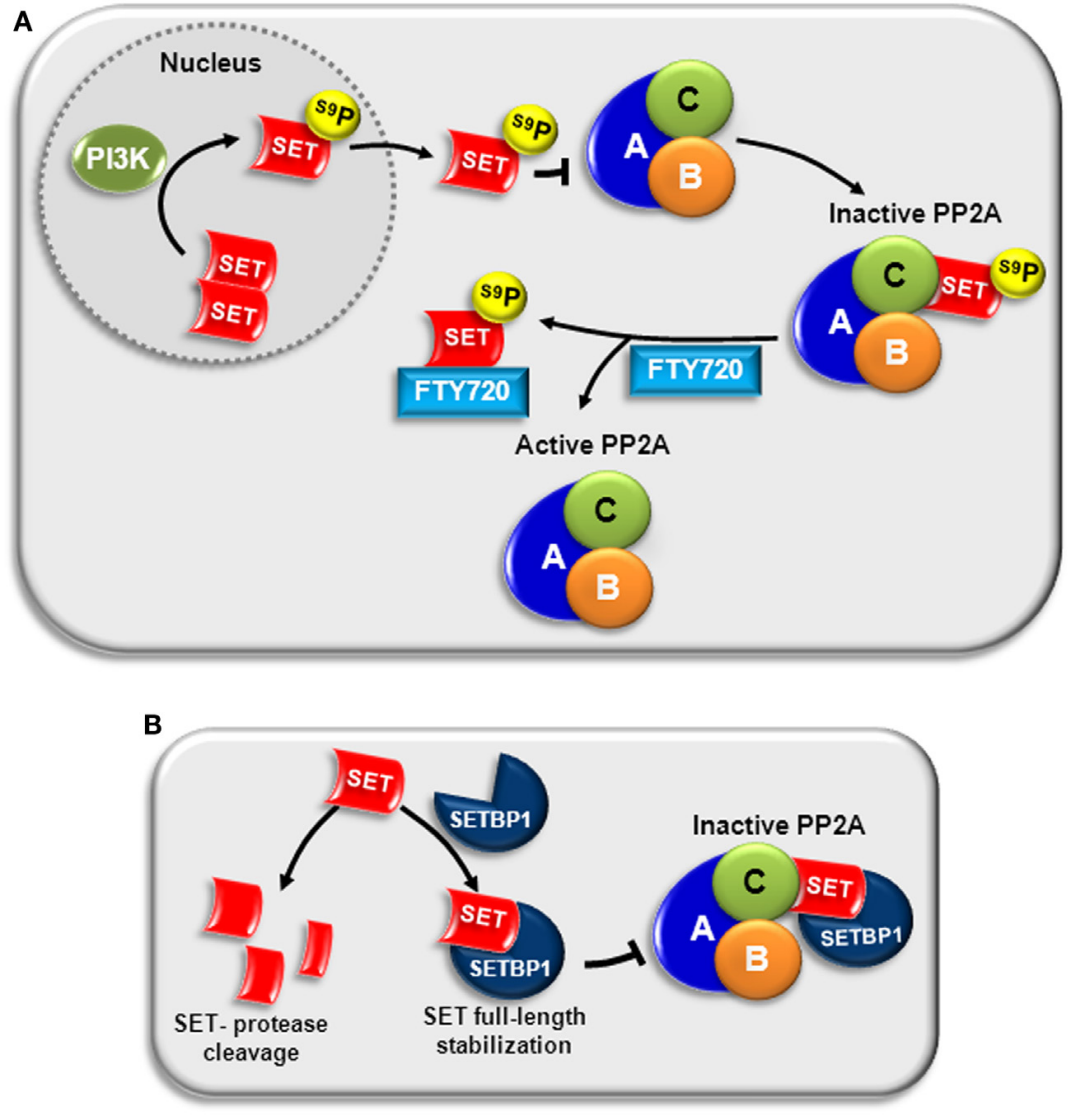
**PP2A inactivation by SET in AML**. **(A)** PI3K can phosphorylate SET at serine 9 (S9), located in the nuclear localization signal. This phosphorylation translocates SET to the cytosol and impairs its return to the nucleus, increasing its ability to bind to the catalytic subunit of PP2A (PP2A-C), and inactivating PP2A (92, 93). Treatment with FTY720 disrupts SET–PP2A interaction, allowing PP2A activation (39). **(B)** SETBP1 binds directly to SET, stabilizing full-length SET and protecting it from protease cleavage. The complex SETBP1–SET binds PP2A through SET, inhibiting PP2A activity (50).

Protein phosphatase 2A is functionally inhibited as a consequence of the overexpression and/or post-translational modifications (e.g., phosphorylation) of SET, which results in an overall inhibition of PP2A phosphatase activity in both leukemic progenitors and stem cells (Figure 2) (13, 28, 29, 38, 85, 86). SET is upregulated in both hematological and solid tumors, including colorectal cancer (87) and breast cancer (88), and in most cases its effects as an oncogene are due to the concomitant inactivation of PP2A (87–90). The role of SET has been studied in depth in chronic myeloid leukemia (CML). SET is overexpressed in CML through BCR-ABL1, the constitutively active oncogenic tyrosine kinase that is essential for CML emergence, maintenance, and progression (85). Expression of BCR-ABL1 allows recruitment and activation of JAK2, which in turn, enhances β-catenin activity and induces SET-mediated inactivation of PP2A (91).

We quantified the expression of *SET* in a series of patients with AML at diagnosis, observing that *SET* overexpression is a recurrent molecular event (60/214, 28%) associated with short overall survival in AML. Interestingly, overexpression of this oncogene also had prognostic impact in patients with normal karyotype, defining a subgroup of patients with a worse outcome. Although multivariate analysis confirmed *SET* overexpression as an independent prognostic marker in our series, it was associated with other adverse prognostic markers, such as monosomy 7, *SETBP1* overexpression, and *EVI1* overexpression. This observation suggests that SET deregulation could cooperate with other additional aberrations in the leukemogenesis program. Analysis by western blot confirmed high SET expression at protein level in both AML cell lines and patient samples. In addition, we observed that SET promotes cell viability by inhibiting the effect of PP2A in AML cells, contributing to malignancy progression (38). We also confirmed in AML cells that SET forms an inhibitory complex with PP2A-C, and that the whole structure of SET (amino-terminus and carboxy-terminus) is involved in the binding (39). Furthermore, the antileukemic effects of FTY720 and OP449, two recently discovered PADs, depend on the interaction/sequestration of SET, pointing out the importance of this oncogene in AML (37, 39). Nevertheless, despite the importance of SET overexpression and its prognostic impact in both hematological and solid tumors, little is known about the mechanisms involved in its transcriptional regulation. In addition, it has been reported that post-translational modification of SET can modulate SET affinity to PP2A. Phosphorylation at serine 9 (S9) in the nuclear localization signal, by either CKII (92) or PI3Kγ (93), impairs its returning to the nucleus, increasing its ability to inactivate PP2A (Figure 2A); whereas S171 phosphorylation by protein kinase D2 (PKD2) decreases its affinity for PP2A, lowering the inhibitory activity (94).

Our group found another mechanism that impairs PP2A activity via SET in AML: the overexpression of *SETBP1* (SET binding protein 1) (50). SETBP1 upregulation leads to binding and stabilization of 39 kDa full-length SET, protecting from protease cleavage. The complex SETBP1–SET binds PP2A through SET, provoking PP2A activity inhibition and promoting cell proliferation. Interestingly, 27% of AML patients have SETBP1 overexpression, and shorter overall survival is predicted in older AML patients with this aberration (50) (Figure 2B). Later studies in other myeloid neoplasms have confirmed the important role of the *SETBP1* oncogene in leukemogenesis.

Interestingly, SET is also implicated in natural killer (NK) cell cytotoxicity. After cytokine stimulation (Interleukin-12, -18, and -15), SET upregulation in human NK impairs IFN-γ production through PP2A inactivation, limiting the anti-tumor and/or anti-inflammatory effect of NK (95). Trotta et al. described a model where SET–PP2A regulates granzyme B expression at mRNA and protein levels, and therefore, determines NK cytotoxicity. They observed that SET knockdown inhibited the induction of granzyme B expression, normally induced by stimulation of NK cells with IL-2 or IL-15, limiting NK cytotoxicity (96). Other reported functions include inhibiting the DNase activity of the tumor-suppressor NM23-H1, increasing AP-1 activity, or activating MAPK signaling (97, 98). These data suggest the role of SET not only as a PP2A inhibitor but also contributing with other signaling pathways to promote tumor growth.

### Cancerous Inhibitor of Protein Phosphatase 2A

Another PP2A endogenous inhibitor is CIP2A (cancerous inhibitor of PP2A) (99). CIP2A controls oncogenic cellular signals by inhibiting PP2A activity toward the oncogenic transcription factor c-MYC (8, 100, 101), which plays an important role in AML (102). CIP2A acts by impairing PP2A activity leading to the stabilization of c-MYC (99).

Cancerous inhibitor of protein phosphatase 2A is expressed in very few normal tissues but it is overexpressed in most human cancer types, where it is often associated with a clinically aggressive behavior (100, 101, 103–106). However, only a few studies have focused on AML. Wang et al., using conventional RT-PCR, found that 77.4% of AML cases expressed CIP2A (55 of 84), and confirmed their results at protein level; however, they provided no quantitative data (107). Recently, our group investigated its prevalence using quantitative real-time RT-PCR in a series of 203 normal karyotype AML patients (NK-AML) at diagnosis, and reported that high CIP2A expression is a is a recurrent event in this AML subgroup (51/203, 25%), with a poor prognostic impact in the overall survival of NK-AML cases (40). Our results indicate that CIP2A behaves as an oncoprotein in AML. CIP2A depletion downregulates c-MYC, leading to a reduction of cell proliferation, supporting the positive relationship between CIP2A and this oncogenic transcription factor in AML. Nevertheless, further studies are needed to elucidate the role of CIP2A in AML.

Cancerous inhibitor of protein phosphatase 2A has been extensively studied in CML. High levels of CIP2A at diagnosis are significantly associated with risk of progressing to blast crisis; therefore, the CIP2A protein level has been proposed as a prospective biomarker of disease progression in imatinib-treated CML patients (108). Moreover, high CIP2A levels are associated with high c-MYC and high BCR-ABL1 tyrosine kinase activity (108). In addition, second-generation tyrosine kinase inhibitors (TKI) disrupt the CIP2A/c-MYC/E2F1 positive feedback loop, leading to lower disease progression risk. These data support that CIP2A inhibits PP2Ac, stabilizing E2F1, and creating a CIP2A/c-MYC/E2F1 positive feedback loop, which imatinib cannot overcome (109).

## PP2A-Activating Drugs

The increased number of studies showing that PP2A is frequently inactivated in cancer and has raised interest in developing new drugs that could act as PP2A activators (12, 110). The most widely studied drugs are FTY720 and OP449.

FTY720 is an oral sphingosine analog derived from myriocin, a metabolite isolated from fungus *Isaria Sinclairii* that has been approved for the treatment of patients with relapsing multiple sclerosis (111). After phosphorylation by sphingosine kinase 2 (SPHK2), FTY720 binds to one of the sphingosine-1-phosphate receptor (S1P_1_, S1P_3_, S1P_4_, or S1P_5_). Phosphorylated FTY720 does not impair T-lymphocyte or B-lymphocyte activation, but does interfere with immune cell trafficking between lymphoid organs and peripheral blood (112).

FTY720 is also a potent inhibitor of tumor growth and angiogenesis, pointing to the use of this drug in the treatment of both solid and hematological tumors. The anticancer activity of FTY720 depends on its ability, at least in leukemias, to act as a potent PP2A activator [reviewed in Ref. (86)]. FTY720-induced PP2A activity induces apoptosis by interfering with BCL2; and suppresses mitogenic and survival signals by inhibiting the ERK and PI3K/AKT pathways (13, 28, 32, 39, 41, 113–115).

The effects induced by FTY720 are well characterized in both Ph positive and negative leukemias. Several reports highlight the efficacy of FTY720 *in vitro* and *in vivo* models of AML, reporting restored PP2A activity, decreased clonogenicity, and suppression of disease (12, 38–41). In CML and Ph-positive B-ALL progenitors, FTY720 promotes BCR-ABL1 inactivation and degradation. This leads to inhibition of survival factors (such as JAK2, AKT, and ERK1/2), which result in apoptosis of CD34+ progenitors in patients with TKI sensitive and TKI-resistant CML (12, 28, 85). These findings are promising in CML and in other myeloproliferative neoplasms, suggesting the possibility that patients could be brought into remission by TKIs and then treated with FTY720 or its derivatives (12).

Mechanistically, as indicated above, FTY720 prevents SET/PP2A-C binding through its interaction with SET in a C-terminal hydrophobic pocket that contains a globular amphipathic domain (116). Our group confirmed these results in AML (39). FTY720, which binds SET within the last 100 amino acids of the C-terminal fragment (39, 117), produces a destabilization of the SET/PP2A-C complex, leading to the reactivation of PP2A function and a reduction of AML cell viability (39). Interestingly, FTY720 not only disrupts complex formation between SET and PP2A-C but it also induces increased translocation of SET to the nucleus (39), possibly by reducing the phosphorylation of SET S9 without affecting the protein levels (86) (Figure 2A). Moreover, apart from inducing apoptosis of AML cells by reactivation of PP2A activity, FTY720 treatment could perturb the sphingolipid metabolism pathway. This disruption leads to the accumulation of ceramide, a pro-apoptotic second messenger, mostly in the mitochondria membrane, contributing to the death of AML cells (115). In addition, in a recent study in *in vitro* and *in vivo* models of AML, we found that FTY720 lipid nanoparticles were more efficient at inducing cell growth arrest and apoptosis than FTY720 in solution in AML cells. Interestingly, the use of lipid nanoparticles containing FTY720 significantly increased oral bioavailability of the free drug. These results provide the first evidence for the potential use of FTY720 lipid nanoparticles as an oral therapeutic agent in AML (41).

Since the anticancer activity of FTY720 does not require SPHK2 phosphorylation or S1PR1 interaction, and FTY720-P seems to have pro-proliferative properties [reviewed in Ref. (12)], it has been proposed that FTY720 analogs that are not targets for phosphorylation by SPHK2, as [S]-FTY720-OMe, [S]-FTY720-regioisomer, and OSU-2S, may have less toxicity and be more useful as anticancer drugs. Of note, these FTY720 derivatives do not induce lymphopenia, undergo phosphorylation, or interact with the S1PR1 receptor [(13), reviewed in Ref. (12)].

Other novel molecules have been tested to activate PP2A in AML, such as the small peptide OP449 (118). OP449 was reported as a novel, physiologically stable, cell-penetrating peptide, which binds specifically to SET and antagonizes the inhibition of PP2A. Furthermore, OP449 treatment suppresses growth, enhances apoptosis, and impairs clonogenicity of CML and AML cell lines and primary patient cells, leading to the activation of the PP2A function. It has been reported that the SET binding peptides COG112 and OP449 reactivate PP2A upon interaction with SET, preventing SET–PP2Ac interaction and, therefore, the inhibition of PP2A activity (90, 118). Furthermore, the combination of OP449 with specific TKI or chemotherapy in treatment of CML and AML cell lines and primary patient samples have synergistic effects (37). These findings open new possibilities to establish innovative strategies for combined therapy that targets PP2A and tyrosine kinase signaling pathways in order to improve therapeutic options in AML patients.

## Conclusion

Despite progressive advances in our understanding of the molecular biology of AML, the general therapeutic strategy in patients with this leukemia has not changed substantially, and the outcome for most patients is poor. New compounds targeting a variety of cellular processes have been developed for the treatment of AML, although few have been translated into clinical practice. Nevertheless, it is unlikely that any of these compounds, when used as single agents, will cure the disease, which suggests the need for combinatorial therapy (7). Furthermore, the results of the Cancer Genome Atlas Research Network confirm the molecular heterogeneity of this disease, and show that genes that are significantly mutated in AML are organized into several functional categories, suggesting the importance of developing treatments directed at target pathways (48). In this regard, the tumor-suppressor PP2A has emerged as a promising therapeutic target in AML, since it is a negative regulator of several survival and proliferation pathways that are frequently activated in AML as a result of aberrant activation of oncogenic kinases.

Protein phosphatase 2A inactivation is a recurrent event in AML, and restoration of its activity by PADs has antileukemic effects in both KIT-positive and KIT-negative AML cells. Preclinical studies show that pharmacological restoration of PP2A tumor-suppressor activity by PADs (e.g., FTY720, FTY720 analogs, or OP499) effectively antagonizes leukemogenesis, and that these drugs have synergistic cytotoxic effects with both conventional chemotherapy and TKIs, opening new possibilities for precision medicine, or personalized treatment, in AML patients (30, 37, 41). Interestingly, the anticancer activity of several PADs depends on interaction/sequestration of its endogenous inhibitor SET, an oncogene overexpressed in 28% of AML patients (38).

These results indicate that the combination of kinase inhibitors and PADs may be a valid therapeutic option for AML, especially for treating leukemias characterized by SET-dependent inactivation of PP2A. Therefore, PADs might be clinically relevant anticancer drugs that could be introduced into therapeutic protocols for patients with hematopoietic and non-hematopoietic malignancies characterized by functional loss of the PP2A tumor suppressor (13, 37).

## Author Contributions

All authors listed, have made substantial, direct, and intellectual contribution to the work, and approved it for publication.

## Conflict of Interest Statement

The authors declare that the research was conducted in the absence of any commercial or financial relationships that could be construed as a potential conflict of interest.

## Abbreviations

ALL, acute lymphoblastic leukemia; AML, acute myeloid leukemia; ATM, ataxia-telangiectasia mutated; CIP2A, cancerous inhibitor of protein phosphatase 2A; CML, chronic myeloid leukemia; LCMT1, leucine carboxyl methyltransferase 1; NK, natural killer; OA, okadaic acid; PADs, PP2A activating drugs; PKR, interferon-inducible dsRNA-activated protein kinase; PP2A, protein phosphatase 2A; SETBP1, SET binding protein 1; SPHK2, sphingosine kinase 2.
